# Severe Mpox Among People With Advanced Human Immunodeficiency Virus Receiving Prolonged Tecovirimat in New York City

**DOI:** 10.1093/ofid/ofae294

**Published:** 2024-05-17

**Authors:** Elizabeth A Garcia, Mary M K Foote, Tristan D McPherson, Maura K Lash, Amma N Bosompem, Alyssa Bouscaren, Justin Chan, Madeline A DiLorenzo, Dennis Feihel, Randal C Fowler, Vani Gandhi, Elizabeth R Jenny-Avital, Erik J Kopping, Dana Mazo, Jacob McLean, Ofole Mgbako, Mark N Sayegh, Raphael N Shaw, Michelle Su, Jeanne Sullivan Meissner, Jade C Wang, Wendy Wen, John C Winters, Cosmina B Zeana, Jason Zucker, Marcia Wong

**Affiliations:** Division of Disease Control, New York City Department of Health and Mental Hygiene, Queens, New York, USA; Division of Disease Control, New York City Department of Health and Mental Hygiene, Queens, New York, USA; Division of Disease Control, New York City Department of Health and Mental Hygiene, Queens, New York, USA; Division of Disease Control, New York City Department of Health and Mental Hygiene, Queens, New York, USA; Human Resources Administration, New York City Department of Social Services, New York, New York, USA; Department of Homeless Services, New York City Department of Social Services, New York, New York, USA; Division of Infectious Diseases and Immunology, New York University Grossman School of Medicine, New York, New York, USA; Department of Internal Medicine and Infectious Diseases, NYC Health + Hospitals/Bellevue, New York, New York, USA; Division of Infectious Diseases and Immunology, New York University Grossman School of Medicine, New York, New York, USA; Lenox Hill Hospital Department of Medicine, Northwell Health, New York, New York, USA; Division of Disease Control, New York City Department of Health and Mental Hygiene, Queens, New York, USA; Division of Infectious Diseases, Icahn School of Medicine at Mount Sinai, New York, New York, USA; Division of Infectious Diseases, NYC Health + Hospitals/Jacobi, Bronx, New York, USA; Division of Disease Control, New York City Department of Health and Mental Hygiene, Queens, New York, USA; Division of Infectious Diseases and Immunology, New York University Grossman School of Medicine, New York, New York, USA; Division of Infectious Diseases, New York Presbyterian Hospital, Columbia University, New York, New York, USA; Division of Infectious Diseases and Immunology, New York University Grossman School of Medicine, New York, New York, USA; Department of Internal Medicine and Infectious Diseases, NYC Health + Hospitals/Bellevue, New York, New York, USA; New York University Langone Institute for Excellence in Health Equity, NYU Langone Health, New York, New York, USA; Division of Infectious Diseases, NYC Health + Hospitals/Harlem, New York, New York, USA; Division of Infectious Diseases, Columbia University, New York, New York, USA; Division of Infectious Diseases, NYC Health + Hospitals/Harlem, New York, New York, USA; Division of Infectious Diseases, Columbia University, New York, New York, USA; Division of Disease Control, New York City Department of Health and Mental Hygiene, Queens, New York, USA; Division of Disease Control, New York City Department of Health and Mental Hygiene, Queens, New York, USA; Division of Disease Control, New York City Department of Health and Mental Hygiene, Queens, New York, USA; Division of Disease Control, New York City Department of Health and Mental Hygiene, Queens, New York, USA; Division of Infectious Diseases, Icahn School of Medicine at Mount Sinai, New York, New York, USA; Department of Internal Medicine and Infectious Diseases, NYC Health + Hospitals/Elmhurst, Queens, New York, USA; Division of Infectious Diseases, BronxCare Health System, Bronx, New York, USA; Division of Infectious Diseases, New York Presbyterian Hospital, Columbia University, New York, New York, USA; Division of Disease Control, New York City Department of Health and Mental Hygiene, Queens, New York, USA

**Keywords:** mpox treatment, HIV/AIDS, tecovirimat, immunocompromise, mortality

## Abstract

Severe mpox has been observed in people with advanced human immunodeficiency virus (HIV). We describe clinical outcomes of 13 patients with advanced HIV (CD4 <200 cells/μL), severe mpox, and multiorgan involvement. Despite extended tecovirimat courses and additional agents, including vaccinia immune globulin, cidofovir, and brincidofovir, this group experienced prolonged hospitalizations and high mortality.

During the 2022 mpox outbreak, 44% of mpox cases in New York City (NYC) from mid-May to mid-August were among people living with human immunodeficiency virus (HIV) (unpublished data). People with advanced HIV have required extended hospitalization for severe mpox manifestations, resulting in significant morbidity and mortality, especially among Black non-Hispanic men [1, 2]. A global case series reported 25% mortality among people with HIV (PWH) with low CD4 counts (CD4 <350 cells/μL) who were hospitalized with mpox [3].

Tecovirimat is approved by the US Food and Drug Administration to treat smallpox and can be used for mpox under an expanded-access Investigational New Drug protocol. Additional mpox investigational agents, including vaccinia immune globulin intravenous (VIG), brincidofovir, cidofovir, and trifluridine, have been used along with tecovirimat as combination therapy for severe mpox. To better inform clinical management, we describe the mpox clinical outcomes among NYC residents living with advanced immunosuppression due to HIV (CD4 <200 cells/μL) receiving prolonged courses of tecovirimat.

## METHODS

This is a retrospective study. Patients with an HIV status known to providers who were living in NYC were included if they had a positive non-variola orthopoxvirus or mpox test, CD4 count <200 cells/μL, and a limited response to tecovirimat (characterized by slow-healing or complex lesions requiring a treatment course >14 days). Cases were collected when healthcare providers contacted the NYC Department of Health and Mental Hygiene (DOHMH) or the Centers for Disease Control and Prevention's (CDC) clinical team for consultation between 2 August 2022 and 16 December 2022.

We analyzed and reviewed DOHMH mpox surveillance for demographic information, any documented clinical information during consultations, and a data collection tool completed by providers. We cross-matched cases by name and date of birth with DOHMH HIV surveillance for CD4 and viral load and NYC Department of Social Services surveillance for housing status. For specimens submitted to the DOHMH Public Health Laboratory with low enough polymerase chain reaction cycle thresholds, whole genome sequencing (WGS) was performed.

The data collection tool included demographic, laboratory, and clinical data, as well as parameters from the Mpox Severity Scoring System (MPOX-SSS) [4], a scoring system designed during the 2022 mpox outbreak to provide a standard measure of mpox disease severity between groups and evaluate treatment response through quantitative standardization. The proposed MPOX-SSS tool dated November 2022 was used for data collection, which has slight variations from the current tool dated June 2023.

## RESULTS

We identified 14 eligible patients; providers returned data tools for 13 patients (Table 1). All patients identified as men; median age was 38 years (range, 26–63 years). Ten (77%) identified as Black, African American, or Afro-Caribbean, 2 (15%) as Hispanic/Latino, and 4 (31%) as other race/ethnicity (providers were able to select all applicable race and ethnicity categories, so percentages add up to >100%). Five (38%) experienced unstable housing in the last year, defined as residing in a shelter, transitional single-room occupancy, or congregate residential housing. Eleven (85%) were reported as not taking antiretroviral therapy (ART) at mpox diagnosis (providers reported if patients were on ART, but no additional adherence data were collected). Median CD4 at the time closest to mpox diagnosis was 32 cells/μL (range, 2–149 cells/μL); 10 (77%) had CD4 counts <50 cells/μL. Two (15%) had received 1 dose of JYNNEOS vaccine, both of whom were diagnosed with mpox within 5 days of vaccination.

**Table 1.** Summary of Demographic Information, Clinical Features, and Outcomes Among People With Human Immunodeficiency Virus With Severe Mpox Manifestations and Extended Tecovirimat Duration^a^ (N = 13)—New York City, 2 August 2022–30 March 2023

Characteristic	Median (Range) or No. (%)
Age, y	38 (26–63)
Gender identity	
Male	13 (100%)
Race/Ethnicity^b^	
Black, including African American or Afro-Caribbean	10 (77%)
Hispanic/Latino	2 (15%)
Asian, including South Asian	1 (8%)
Native Hawaiian or Pacific Islander	1 (8%)
Does not identify as any of these races	2 (15%)
Housing status	
Unstably housed in the past year^c^	5 (38%)
HIV-related characteristics	
CD4 count, cells/μL	32 (2–149)
<50	10 (77%)
50–200	3 (23%)
Viral load, copies/mL	55 726 (74–870 964)
<200	1 (8%)
200–1000	0 (0%)
1001–100 000	9 (69%)
>100 000	3 (23%)
Not on ART at time of mpox diagnosis	11 (85%)
JYNNEOS status^d^	
Did not receive JYNNEOS vaccine	11 (85%)
Received 1 dose of JYNNEOS vaccine	2 (15%)
Received 2 doses of JYNNEOS vaccine	0 (0%)
Length of time between mpox symptom onset and tecovirimat initiation, d	14.5 (0–63)
Length of time between date of mpox specimen collection and tecovirimat initiation, d	3.5 (0–22)
Tecovirimat formulation	
Oral only	4 (31%)
Oral and IV	9 (69%)
Tecovirimat duration of treatment, d	58.5 (22–133)
Tecovirimat adverse reactions reported	0 (0%)
Coadministered medications	
VIG	7 (54%)
Multiple doses of VIG	5 (38%)
Cidofovir	5 (38%)
Intralesional/topical cidofovir	1 (8%)
Brincidofovir	4 (31%)
Trifluridine eye drops	3 (23%)
WGS results	
Specimens sequenced	9 (69%)
F13L gene mutation detected	4 (44%)
Hospitalized for mpox	12 (92%)
Reason for hospitalization^b^	
Confluent or severe skin lesions	10 (77%)
Disseminated disease requiring ICU admission	7 (54%)
Severe bacterial superinfection	7 (54%)
Pain control	5 (38%)
Inability to swallow	1 (8%)
Inability to isolate/social reasons	1 (8%)
Length of hospitalization, d	56 (8–203)
Received treatment for pain	12 (92%)
Received steroids	5 (38%)
Received pressors	5 (38%)
Intubated	4 (31%)
Surgery required	4 (31%)
Other infections reported	
Bacteremia	3 (23%)
Sepsis	2 (25%)
Skin/soft tissue infections	7 (54%)
Osteomyelitis	1 (8%)
Other complications	
Bleeding	2 (15%)
Worsening kidney function	5 (38%)
Worsening liver function	3 (23%)
Cavitary pulmonary lesions	3 (23%)
Neurologic complications	1 (8%)

Abbreviations: ART, antiretroviral therapy; ICU, intensive care unit; IV, intravenous; PWH, people with human immunodeficiency virus; VIG, vaccinia immune globulin intravenous; WGS, whole genome sequencing.

^a^Tecovirimat course >14 days.

^b^Multiple categories could be selected; percentages may add up to >100%.

^c^Unstably housed defined as shelter, transitional single room occupancy, or congregate residential housing.

^d^All recipients of JYNNEOS were diagnosed with mpox within 5 days of receiving the vaccine. JYNNEOS was not administered as postexposure prophylaxis.

Median time from mpox symptom onset to tecovirimat initiation was 14.5 days (range, 0–63 days). Median time from date of mpox testing to tecovirimat initiation was 3.5 days (range, 0–22 days). The median duration of tecovirimat treatment was 58.5 days (range, 22–133 days). Other medications coadministered with tecovirimat included VIG (n = 7 [54%]), cidofovir (n = 5 [38%]), brincidofovir (n = 4 [31%]), and trifluridine eye drops for ocular involvement (n = 3 [23%]). Five patients received multiple doses of VIG. One patient received intralesional/topical cidofovir. Two patients experienced acute kidney injury, and 1 patient experienced worsening liver function, following cidofovir administration. One patient experienced hepatotoxicity following brincidofovir administration. All patients received ART during hospitalization.

One patient (8%) was treated as an outpatient and had lesion resolution. Twelve patients (92%) were hospitalized with severe mpox manifestations; the median length of hospitalization was 56 days (range, 8–203 days). Severe manifestations included necrotic facial lesions, bacterial superinfection of the eye, ocular collapse, cavitary pulmonary lesions, airway edema requiring intubation and tracheostomy, esophageal ulcers, and uncontrollable rectal bleeding. Reasons for hospitalization, other infectious disease manifestations, and other complications are listed in Table 1. Among hospitalized patients, 6 patients (46%) died, and 6 were discharged (46%).

MPOX-SSS scores could be calculated for 9 patients (69%). The median severity score at initial presentation was 12 (range, 8–17), and the median final severity score upon discharge or death was 10 (range, 1–22). Among 4 patients who died, the median initial severity score was 16 (range, 9–17), and the median final severity score was 21 (range, 17–22). Among 5 patients whose lesions resolved, the median initial severity score was 12 (range, 8–17), and the median final severity score was 3 (range, 1–10) (Supplementary Figure 1).

Specimens from 11 (85%) patients were submitted to the Public Health Laboratory for analysis. Specimens were sequenced for 9 of these patients; specimens for 2 patients failed to meet quality endpoints required for WGS. Of these 9 specimens, 4 (44%) had a mutation in the F13L gene associated with tecovirimat resistance.

## DISCUSSION

This group of patients living with advanced HIV and mpox experienced prolonged hospitalization and high mortality, which is consistent with previous literature [1, 3, 5–7]. Nearly half of the patients in this case series died. Most identified as Black, were unstably housed, and were disengaged from HIV care. Notable manifestations of severe mpox included ocular involvement culminating in globe collapse, gastrointestinal hemorrhage, and disfiguring necrotic facial lesions. All received extended tecovirimat treatment courses. Tecovirimat is not viricidal, and effective immune responses are required to clear mpox infection [8]. Early initiation of ART in these patients, and rapid and multipronged mpox treatment approach, including tecovirimat in combination with VIG, cidofovir, and/or brincidofovir, may improve clinical outcomes [9–11]. Studies to assess the efficacy of tecovirimat for mpox are underway, but additional data are needed to understand the impact of combination therapy.

Broader access to tecovirimat resistance testing is needed to guide clinical decision making in this population. Four percent of CDC-sequenced specimens had F13L mutations that may indicate resistance [9], with almost 60% of those having confirmed phenotypic resistance, all found in PWH [12]. In our case series, which was limited to patients who received extended tecovirimat courses, 9 patients had specimens that underwent sequencing, of which 4 (44%) specimens had F13L genotypic mutations; however, without paired phenotypic results, these data are not conclusive for resistance. There may be an increased risk of tecovirimat resistance in this population due to severe immunocompromised status and extended treatment duration, as well as oral absorption concerns that may lead to subtherapeutic concentrations [13]. In the absence of resistance testing, we suggest a low threshold to switch to intravenous tecovirimat to increase bioavailability, and early combination therapy with other antivirals [9].

Previous case series highlighting mpox severe outcomes and death have raised concerns for immune reconstitution inflammatory syndrome (IRIS) [3]. IRIS was generally not suspected by clinicians in this case series due to sustained low CD4 counts and continued immunosuppression despite initiation of ART during hospitalization for most patients, with severe outcomes possibly resulting from progression of mpox and overwhelming and unchecked viral replication [8]. Prompt ART initiation in PWH not on treatment at the time of mpox diagnosis is a critical component of mpox treatment, as optimized immune responses are essential to clear viral infection [8, 9, 14].

This case series applies the MPOX-SSS to severe cases. While data were only available for 69% of patients, patients who died had a higher median initial severity score that increased over time, compared to lower initial severity scores that improved over time in those who survived. While additional validation is needed, these trends suggest that MPOX-SSS could be a useful tool in triaging severe mpox cases and assist in decision making around medical countermeasures.

This group faced intersectional barriers to HIV care and mpox prevention, as well as delays in presentation to care following mpox symptom onset, which may lead to mpox progression [15]. Strategies to engage and retain PWH in care require mitigation of structural barriers to accessing and sustaining engagement in care, including linkage to social support services such as housing, as well as proactive outreach to PWH disengaged from care [16–20]. Mpox vaccination and preventive care efforts should ensure that structural racism and institutional inequities are addressed [21–23].

Detailed clinical courses for several patients in this case series have been published [24, 25]. Strengths of this case series include cross-matching clinical data with NYC public health surveillance and social service data systems, reporting of WGS results, and use of the MPOX-SSS to characterize severe mpox manifestations. Potential limitations include dependence on provider reporting to identify cases based upon requests for VIG and other medical countermeasures; convenience sampling that may not be representative of all severe mpox cases in NYC or among people with advanced HIV with CD4 counts <200 cells/μL; and lack of phenotypic resistance testing results.

## CONCLUSIONS

These mpox patients with advanced HIV experienced high social vulnerability, severe mpox manifestations, and high mortality. Factors associated with poor HIV outcomes may contribute to severe mpox outcomes, necessitating proactive outreach, vaccination, and other preventive measures for this population. ART for HIV, as well as early and extended tecovirimat with coadministration of other mpox treatments, is essential to improving clinical outcomes from mpox. Findings of severe disease and high mortality highlight the urgency of mitigating deep social inequities and the need for research to optimize mpox prevention and care among PWH.

## Supplementary Material

ofae294_Supplementary_Data

## Supplementary Data

Supplementary materials are available at *Open Forum Infectious Diseases* online. Consisting of data provided by the authors to benefit the reader, the posted materials are not copyedited and are the sole responsibility of the authors, so questions or comments should be addressed to the corresponding author.

## Notes

***Acknowledgments.*** We thank members of the New York City (NYC) Public Health Laboratory Virology, Whole Genome Sequencing, and Bioinformatics units for their roles in coordinating mpox lesion specimen testing and genomic analyses. The authors acknowledge the inspiring care, compassion, and strength of our NYC provider community, who confronted a multitude of challenges during this outbreak. We would also like to acknowledge the patients and their families who still sought to contribute to our knowledge in the face of incredible suffering and amidst the uncertainty of a reemerging disease.

***Author contributions.*** E. A. G. and M. W. contributed to the design of the case series and contributed to the data collection, data analysis, data interpretation, and drafting and revision of the manuscript. M. K. L., T. D. M., and M. M. K. F. contributed to the design of the case series and contributed to the data collection, data interpretation, and drafting and revision of the manuscript. W. W., J. S. M., A. N. B., A. B., E. J. K., M. S., R. C. F., O. M., M. A. D., D. M., J. C., J. Z., J. M., C. B. Z., D. F., R. N. S., M. N. S., J. C. Wa., J. C. Wi., E. R. J.-A., and V. G. contributed to the data collection and revision of the manuscript. All authors reviewed and approved the final manuscript.

***Patient consent.*** This case series does not include factors necessitating patient consent. The design of this case series has been approved by the NYC Department of Health and Mental Hygiene's Institutional Review Board as exempt human subjects research under 45 Code of Federal Regulations 46.1104(d)(4)(ii) and (iii).

***Financial support.*** J. Z. and J. M. are supported by the National Institutes of Health (grant numbers K23AI150378 and 5T32AI100852-10, respectively).
