# Supplementary material for: Severe Mpox Among People With Advanced Human Immunodeficiency Virus Receiving Prolonged Tecovirimat in New York City
Source: Open Forum Infect Dis. 2024 May 17;11(6):ofae294. doi: 10.1093/ofid/ofae294 (PMC11168585; doi:10.1093/ofid/ofae294)
Supplement: ofae294_Supplementary_Data [file ofae294_supplementary_data.docx]

### SUPPLEMENTARY DATA

**Supplemental Figure 1: MPOX-SSS scores among patients with lesion resolution (n=5) and among patients who died (n=4) for whom severity scores could be calculated—New York City, August 2, 2022–March 30, 2023**

MPOX-SSS = Mpox Severity Scoring System. Providers were asked to provide MPOX-SSS scores for four time points: Tecovirimat initiation: severity at time of tecovirimat treatment initiation; Clinical Escalation 1: hospitalization after outpatient tecovirimat initiation, starting additional mpox treatment, transferring from floor to ICU, or other; Clinical Escalation 2: hospitalization after outpatient tecovirimat initiation, starting additional mpox treatment, transferring from floor to ICU, or other; Final: current mpox severity or severity at last follow-up. All four time points were not available for every patient. Severity scores were calculated if at least two time points were available.


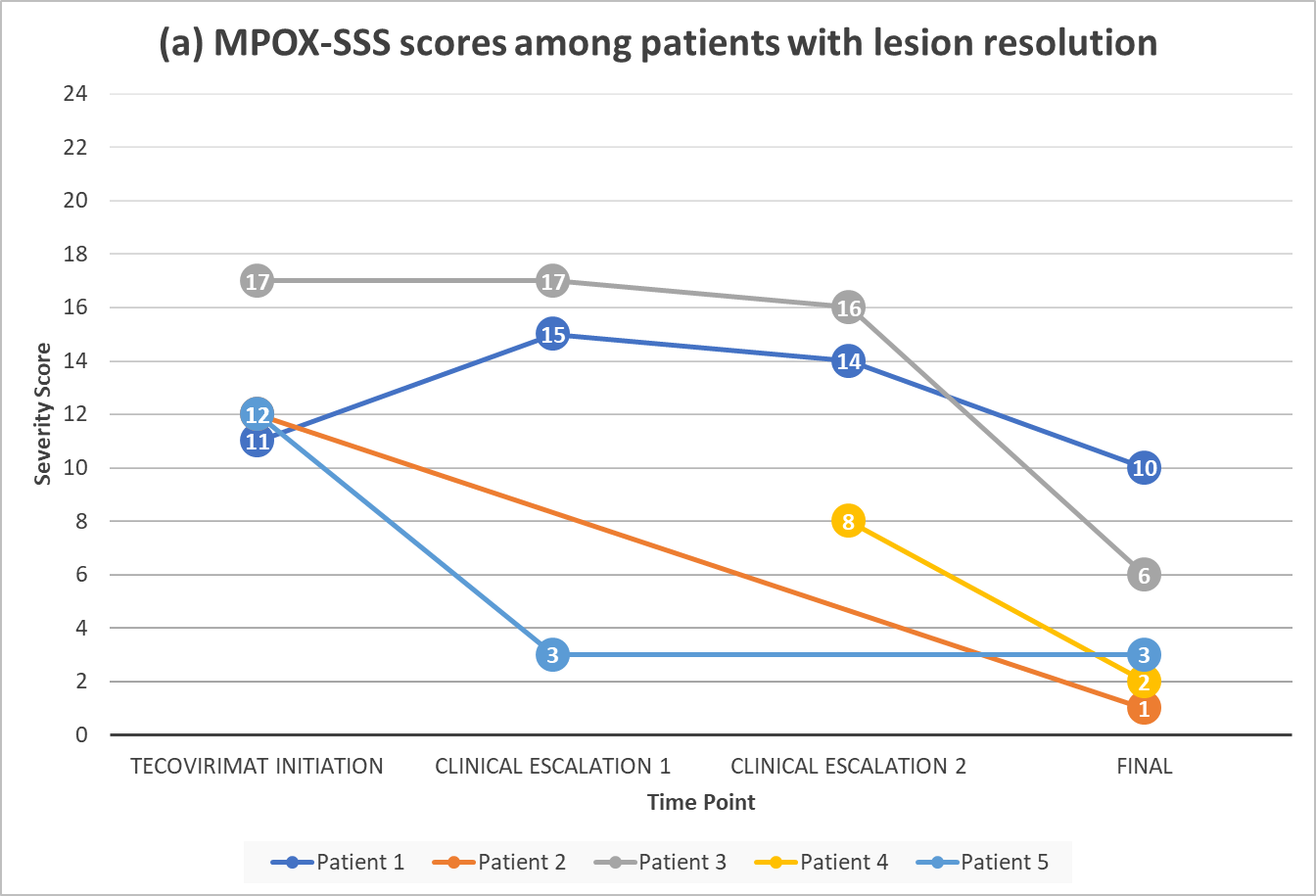


1. Mpox severity scores among patients with lesion resolution (n=5) at four time points (tecovirimat escalation, clinical escalation 1, clinical escalation 2, final).


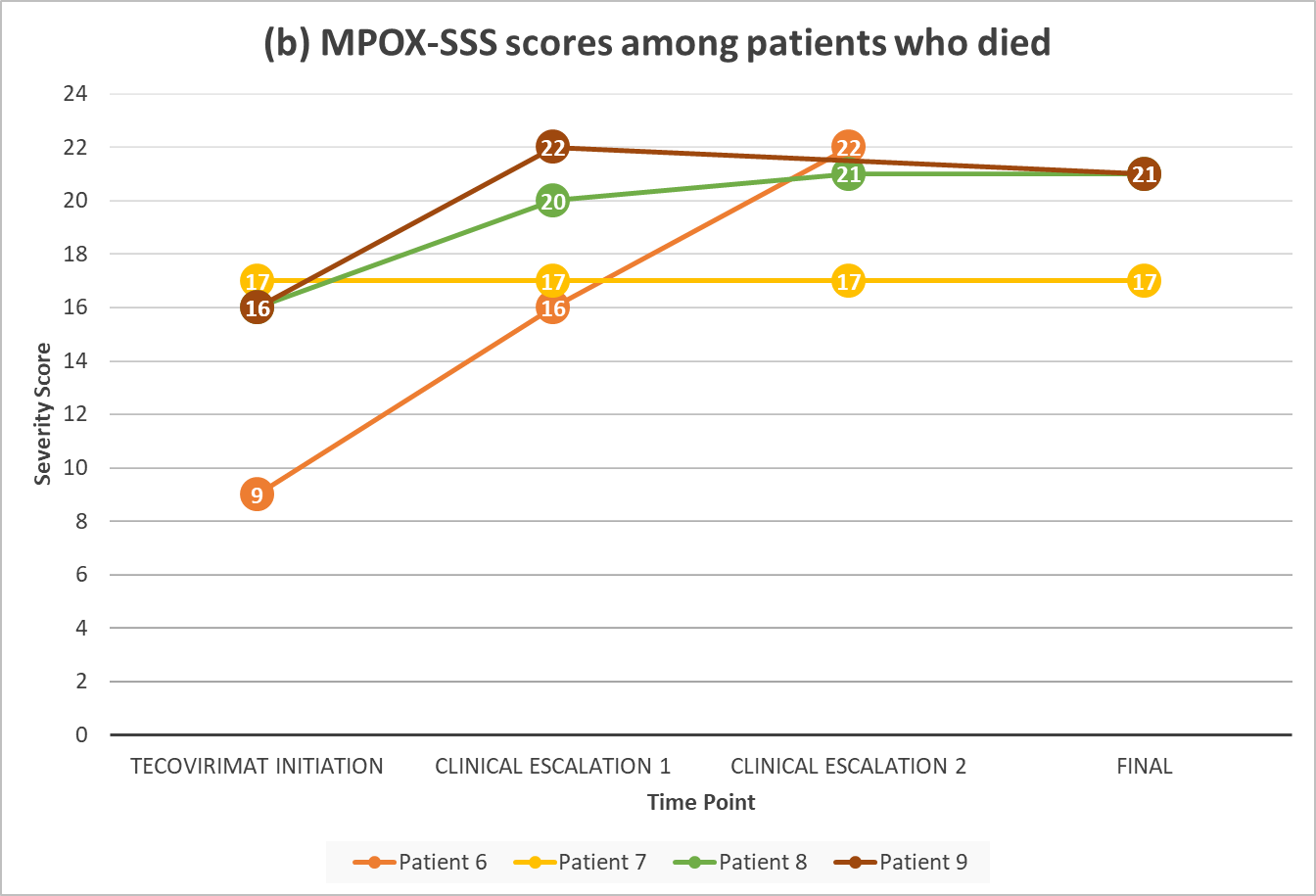


1. Mpox severity scores among patients who died (n=4) at four time points (tecovirimat escalation, clinical escalation 1, clinical escalation 2, final).
